# Predicting gamma evaluation results of patient‐specific head and neck volumetric‐modulated arc therapy quality assurance based on multileaf collimator patterns and fluence map features: A feasibility study

**DOI:** 10.1002/acm2.13622

**Published:** 2022-05-18

**Authors:** Sangutid Thongsawad, Somyot Srisatit, Todsaporn Fuangrod

**Affiliations:** ^1^ Department of Nuclear Engineering Faculty of Engineering Chulalongkorn University Bangkok Thailand; ^2^ Princess Srisavangavadhana College of Medicine Chulabhorn Royal Academy Bangkok Thailand

**Keywords:** gamma prediction, machine learning, patient‐specific VMAT QA

## Abstract

The purpose of this study was to develop a predictive model for patient‐specific VMAT QA results using multileaf collimator (MLC) effect and texture analysis. The MLC speed, acceleration and texture analysis features were extracted from 106 VMAT plans as predictors. Gamma passing rate (GPR) was collected as a response class with gamma criteria of 2%/2 mm and 3%/2 mm. The model was trained using two machine learning methods: AdaBoost classification and bagged regression trees model. GPR was classified into the “PASS” and “FAIL” for the classification model using the institutional warning level. The accuracy of the model was assessed using sensitivity and specificity. In addition, the accuracy of the regression model was determined using the difference between predicted and measured GPR. For the AdaBoost classification model, the sensitivity/specificity was 94.12%/100% and 63.63%/53.13% at gamma criteria of 2%/2 mm and 3%/2 mm, respectively. For the bagged regression trees model, the sensitivity/specificity was 94.12%/91.89% and 61.18%/68.75% at gamma criteria of 2%/2 mm and 3%/2 mm, respectively. The root mean square error (RMSE) of difference between predicted and measured GPR was found at 2.44 and 1.22 for gamma criteria of 2%/2 mm and 3%/2 mm, respectively. The promising result was found at tighter gamma criteria 2%/2 mm with 94.12% sensitivity (both bagged regression trees and AdaBoost classification model) and 100% specificity (AdaBoost classification model).

## 1 INTRODUCTION

Modern radiation therapy techniques, such as volumetric‐modulated arc therapy (VMAT), have been clinically used to achieve radiation therapy goals. VMAT is one of the most common techniques used to treat cancer patients given its local control, ability to prevent dosage to normal tissue, and the ability for dose escalation.^1^, ^2^, ^3^, ^4^, ^5^ VMAT is performed by varying the dose rate, gantry speed, and multileaf collimator (MLC) position during gantry rotation. Before beam delivery, the medical physicist should ensure the linear accelerator machine (linac) can deliver the dose or beam corresponding to the prescribed plan, namely conducting patient‐specific quality assurance (QA). Patient‐specific QA can be performed with different methods such as measurement‐based with a phantom, electronic portal imaging device (EPID)‐based dosimetry with or without a phantom, and log‐file analysis where information is directly retrieved from the linac.

The agreement between beam delivery and dose calculation in treatment planning system (TPS) can be determined by using various methods such as gamma analysis,^6^, ^7^ dose difference,^8^ distance‐to‐agreement (DTA),^9^, ^10^ and dose–volume histogram (DVH) analysis^11^, ^12^, ^13^ in 3D dose distribution. Gamma analysis is shown as the widely used method to determine agreement by setting the criteria of distance and dose difference together. The results of an agreement are shown as gamma passing rate (GPR), which is the ratio between the number of measurements passing (i.e., meeting the criteria) to the total number of measurements. American Associated Physics in Medicine Task Group No. 218 (AAPM Task Group No. 218)^14^ recommended using gamma analysis to evaluate patient‐specific QA with gamma criteria of 3%/2 mm for general purpose and tighter criteria for detecting subtle regional errors. The practical drawbacks of patient‐specific QA have been reported,^15^, ^16^, ^17^, ^18^ such as time‐consuming measurement, resource‐intensive, patient scheduling impact, repeated measurement in case of fail QA. To solve the drawback of patient‐specific QA, many publications have developed a model‐based to predict GPR results with different purposes; to reduce the iterative process of patient‐specific QA,^19^, ^20^ the possibility of replacing the traditional QA process.^21^

One of the significant factors that impact the patient‐specific VMAT QA results is the plan complexity. A plan that contains higher complexity may produce more uncertainty in beam delivery and result in a lower GPR. This complexity is caused by the monitor unit (MU), deviation of the beam intensity, leaf position and trajectory, and aperture area. Many research groups have proposed a score to determine the plan complexity. For example, the modulation index (MI) was the first complexity score introduced by Webb et al.^22^ It was used to determine the beam complexity based on the mean and standard deviation (SD) of the beam fluence. McNiven et al.^23^ introduced the modulation complexity scores (MCS) calculated from the variability of leaf positions, aperture area between segments, and segment weight.

In addition, the correlation between beam complexity scores and QA results were investigated. Rajasekaran et al.^24^ evaluated the correlation between beam complexity matrices and GPR using commercial diode arrays. The global and local gamma indexes showed a weak correlation to the MCS. However, Park et al.^25^ modified MI based on the speed and acceleration of MLC movement, and the correlation between modified MI and QA results was determined. The resulting GPR of patient‐specific VMAT QA were positive. A similar finding was demonstrated by Masi et al.^26^; the high‐intensity modulation involved in the MLC movements and frequent use of small or irregular fields can affect the beam delivery accuracy. Wang et al.^27^ evaluated the correlation between GPR and the IMRT plan's complexity due to MLC position errors. The observed susceptibility was independent of the delivery technique. Park et al.^28^ determined a new metric of MLC speeds and accelerations to predict the plan delivery accuracy of VMAT, with strong correlations to VMAT delivery accuracy. Park et al.^29^ also studied the correlation between image textures of planned fluences and GPR measured from MapCHECK2 and ArcCHECK dosimeters, demonstrating that image textures strongly correlated with the global GPR. Thus, image textures from planned fluence can estimate the VMAT delivery accuracy without processing the patient‐specific QA.

This work proposes a method for predicting patient‐specific VMAT QA results in head and neck patients based on the features extracted from MLC patterns and the fluence map from the plan using a machine learning algorithm. This method improved the treatment planning process using an additional quick step for plan quality assessment. The predictive model of patient‐specific VMAT QA results in head and neck patients were developed using historical GPR of clinical EPID dosimetry without a phantom. The supervised learning features were directly extracted from the treatment plan, including MLC patterns from both banks and the 2D fluence map generated from each delivery arc. The ensemble of trees machine learning in classification and regression models were tested to determine the optimal predictive model based on sensitivity and specificity scores in the testing environment. The proposed method will reduce the risk of the re‐optimization planning process if the plan fails the patient‐specific QA by identifying the failure results prior to measurement.

## 2 MATERIAL AND METHODS

### 2.1 Clinical data collection

One hundred and six VMAT plans (a total of 268 arcs) of the head and neck were randomly collected over a time period from 2018 to 2019 from a single center. All plans were treated with 6 MV photons. Table 1 shows the plan information. The simultaneous integrated boost (SIB) technique was used with different dose prescriptions according to the staging and type of tumor. Dose prescription can be categorized into six prescription protocols. The first protocol was prescribed with 70 Gy (2.12 Gy × 33 fractions) for a gross tumor, 59.4 Gy (1.8 Gy × 33 fractions) for high‐risk nodes, and 54 Gy (1.64 Gy × 33 fractions) for low‐risk nodes. The second protocol was prescribed with 70 Gy (2.12 Gy × 33 fractions) for a gross tumor and 59.4 Gy (1.8 Gy × 33 fractions) for high‐risk nodes. The third protocol was prescribed with 70 Gy (2.12 Gy × 33 fractions) for a gross tumor, 66 Gy (2 Gy × 33 fractions) for high‐risk nodes, 59.4 Gy (1.8 Gy × 33 fractions) for intermediate‐risk nodes, and 54 Gy (1.64 Gy × 33 fractions) for low‐risk nodes. The fourth protocol was prescribed with 70 Gy (2 Gy × 35 fractions) for a gross tumor and 59.5 Gy (1.7 Gy × 35 fractions) to high‐risk nodes. The fifth protocol was prescribed with 70 Gy (2 Gy × 35 fractions) for a gross tumor and 63 Gy (1.8 Gy × 35 fractions) for high‐risk nodes. The sixth protocol was prescribed with 56 Gy (2 Gy × 28 fractions) for a gross tumor. The range of arc numbers for the VMAT plan was 2–4 arcs per plan.

**TABLE 1:** Summary of the randomly selected VMAT head and neck plans used

Tumor region (*n* = plan)
Nasopharynx (*n* = 84)
Supraglottic (*n* = 6)
Floor of mouth (*n* = 2)
Tongue (*n* = 2)
Base of tongue (*n* = 2)
Neck nodes (*n* = 2)
Tonsil (*n* = 2)
Glottis and thyroid (*n* = 2)
Thyroid (*n* = 2)
Buccal (*n* = 2)

Dose prescription (*n* = plan)
First protocol (*n* = 74)
Second protocol (*n* = 18)
Third protocol (*n* = 8)
Fourth protocol (*n* = 2)
Fifth protocol (*n* = 2)
Sixth protocol (*n* = 2)

**Total (*n* = 106)**

### 2.2 Linac, treatment planning system, and EPID‐based dosimetry

Patient‐specific VMAT QA was performed for all plans before treatment using an EPID dosimetry technique. All selected plans were delivered with a TrueBeam linear accelerator (Varian Medical Systems, Palo Alto, CA). The planned 2D dose distribution at the EPID level (source–imager distance = 100 cm) was generated using Portal Dose Image Prediction (PDIP) from the Eclipse TPS (Version 13.6, Varian Medical Systems, Palo Alto, CA). The beam delivery data as an integrated EPID image per arc was collected from aSi‐1000 EPID, a spatial resolution of 1024 × 768 pixels with a pixel size spacing of 0.39 mm. These measured EPID images were then used to compare the planned EPID images generated from PDIP in TPS using the 2D gamma evaluation method. In this study, gamma evaluation was determined using two sets of criteria based on recommendations of AAPM Task Group No. 218.^14^ The first criteria was 2%/2 mm with a 10% global aperture threshold for detecting subtle regional errors, and the second criteria was 3%/2 mm with a 10% global aperture threshold for general purpose. The outcome of this comparison was GPR.

### 2.3 Overall process and feature extraction

**FIGURE 1:**
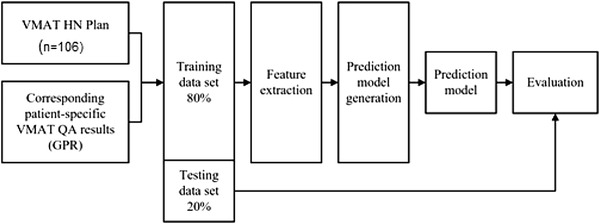
Flow chart diagram for this study

Figure 1 demonstrates the process used in this study. There were 106 VMAT of head and neck plans randomly retrieved. Eighty percent of the total plans (214 arcs) were used as a training dataset and the remaining 20% of the total plans (54 arcs) were used as the testing dataset. DICOM‐RT plan was used to extract the features, including leaf speed, leaf acceleration, and fluence texture. These features were set as the predictor, and the GPR related to the plan was set as the response. The input from extracted features and corresponding GPR were used for generating the predictive model. The evaluation method was performed to determine the accuracy of the prediction model using a testing dataset. All processes were implemented in MATLAB software version 2019b (The Mathworks, Inc, Natick, MA) with machine learning Toolbox 11.6 (classification learner and regression learner application).

Table 2 shows the list of features used for training the model that can be classified into two main groups: (1) features of leaf speeds and accelerations and (2) texture analysis from the fluence map. Leaf speed and acceleration were calculated according to the study of Park et al. ^30^ Leaf speed and acceleration can be calculated using Equations (1) and (2), respectively.

(1) $$\;\text{Leaf}\;\text{spee}d_{i}=\frac{\mathrm{Lea}f_{i}+\mathrm{Lea}f_{i+1}}{\mathrm{Tim}e_{i}}\;.$$

**TABLE 2:** Summary of the features used for this study

Leaf speed and acceleration (Bank A and B)
1) Max. LS Bank A, Max.LS Bank B
2) Mean LS Bank A, Mean LS Bank B
3) SD. LS Bank A, SD. LS Bank B
4) LS_0‐4_ Bank A, LS_0‐4_ Bank B
5) LS_4‐8_ Bank A, LS_4‐8_ Bank B
6) LS_8‐12_ Bank A, LS_8‐12_ Bank B
7) LS_12‐16_ Bank A, LS_12‐16_ Bank B
8) LS_16‐20_ Bank A, LS_16‐20_ Bank B
9) Max. LA Bank A, Max. LA Bank B
10) Mean LA Bank A, Mean LA Bank B
11) SD. LA Bank A, SD. LA Bank B
12) LA_0‐40_ Bank A, LA_0‐40_ Bank B
13) LA_40‐80_ Bank A, LA_40‐80_ Bank B
14) LA_80‐120_ Bank A, LA_80‐120_ Bank B
15) LA_120‐160_ Bank A, LA_120‐160_ Bank B
16) LA_160‐200_ Bank A, LA_160‐200_ Bank B
Texture analysis
17) Contrast
18) Correlation
19) Energy
20) Entropy
21) Homogeneity

Abbreviations: LA, leaf acceleration; LA*_n_*_1−_ *_n_*_2_, leaf acceleration fraction at range between *n*1 to *n*2 mm/s^2^; LS, leaf speed; LS*_n_*_1−_ *_n_*_2_, leaf speed fraction at range between *n*1 to *n*2 mm/s; Max., maximum; SD, standard deviation.

Leaf*_i_* is the position of the leaf at the *i*th CP, and Time*_i_* is the time between the *i*th CP and (*i*+1)th CP. In this study, the time between CP was 0.424 s, which was calculated from the relation between gantry speed of 4.8 deg/s and the interval CP of 2.0341^°^ (178 CPs/full).

(2) $$\;\text{Leaf}\;\text{acceleratio}n_{i}=\frac{\text{Leaf}\;\text{spee}d_{i}+\text{Leaf}\;\text{spee}d_{i+1}}{\mathrm{Tim}e_{i}}\;.$$

The leaf speed and acceleration's extracted features were maximum (Max. LS/LA Bank A, Max. LS/LA Bank B), mean (Mean. LS/LA Bank A, Mean. LS/LA Bank B), standard deviation (SD LS/LA Bank A, SD. LS/LA Bank B), and a fraction of leaf speed and acceleration in different ranges. Since no leaf speed more than 20 mm/s was observed in VMAT plans of this study, the fraction of leaf speed was counted from 0 to 4 mm/s (LS_0‐4_ Bank A, LS_0‐4_ Bank B), from 4 to 8 mm/s (LS_4‐8_ Bank A, LS_4‐8_ Bank B), from 8 to 12 mm/s (LS_8‐12_ Bank A, LS_8‐12_ Bank B), from 12 to 16 mm/s (LS_12‐16_ Bank A, LS_12‐16_ Bank B), and from 16 to 20 mm/s (LS_16‐20_ Bank A, LS_16‐20_ Bank B). Similar to leaf speed, since no leaf acceleration more than 200 mm/s^2^ was observed in VMAT plans of this study, the fraction of leaf acceleration was counted from 0 to 40 mm/s^2^ (LA_0‐40_ Bank A, LA_0‐40_ Bank B), from 40 to 80 mm/s^2^ (LA_40‐80_ Bank A, LA_40‐80_ Bank B), from 80 to 120 mm/s^2^ (LA_80‐120_ Bank A, LA_80‐120_ Bank B), from 120 to 160 mm/s^2^ (LA_120‐160_ Bank A, LA_120‐160_ Bank B), and from 160 to 200 mm/s^2^ (LA_160‐200_ Bank A, LA_160‐200_ Bank B). Park's method^31^ was implemented to generate a gray‐level co‐occurrence matrix (GLCM) for texture analysis. The MLC data and MU for each control point were reconstructed using the integrated intensity fluence map by accumulating all control point fluence maps, then GLCM was generated for each arc. In this study, the particular displacement distances (*d*) of 1 pixel, and four angles (θ) of 0°, 45°, 90°, and 135° were used to calculate the GLCM. The texture analysis's extracted features were contrast, correlation, energy, entropy, and homogeneity.

Leaf speed and acceleration were collected as the predictor for this study because a previous study by Park et al.^20^ showed a strong correlation of leaf speed and acceleration to GPR with a range between −0.458 to −0.511 for leaf speed fraction and −0.225 to 0.477 for leaf acceleration fraction. Additional features collected in this study were texture analysis parameters because another study by Park et al.^21^ showed a strong correlation in the range of −0.475 and 0.213. Only displacement distances (*d*) of 1 pixel were used in this study because a previous study^21^ showed the best correlation at 1 pixel.

The average and SD of features used in this study are shown in Table 3.

**TABLE 3:** Mean and SD of features used for this study

Features	Mean ± SD
Max. LS Bank A	19.59 ± 0.38 mm/s
Mean LS Bank A	10.97 ± 1.04 mm/s
SD. LS Bank A	7.18 ± 0.20 mm/s
LS_0‐4_ Bank A	0.63 ± 0.07
LS_4‐8_ Bank A	0.09 ± 0.03
LS_8‐12_ Bank A	0.05 ± 0.01
LS_12 ‐16_ Bank A	0.03 ± 0.01
LS_16‐20_ Bank A	0.19 ± 0.05
Max. LS Bank B	19.59 ± 0.38 mm/s
Mean LS Bank B	11.36 ± 1.03 mm/s
SD. LS Bank B	7.27 ± 0.20 mm/s
LS_0‐4_ Bank B	0.60 ± 0.07
LS_4‐8_ Bank B	0.09 ± 0.03
LS_8‐12_ Bank B	0.05 ± 0.01
LS_12 ‐16_ Bank B	0.03 ± 0.01
LS_16‐20_ Bank	0.21 ± 0.05
Max. LA Bank A	46.94 ± 0.87 mm/s^2^
Mean LA Bank A	15.63 ± 1.01 mm/s^2^
SD. LA Bank A	13.24 ± 1.06 mm/s^2^
LA_0‐40_ Bank A	0.56 ± 0.09
LA_40‐80_ Bank A	0.10 ± 0.04
LA_80‐120_ Bank A	0.04 ± 0.01
LA_120 ‐160_ Bank A	0.09 ± 0.03
LA_160‐200_ Bank A	0.21 ± 0.04
Max. LA Bank B	46.90 ± 0.87 mm/s^2^
Mean LA Bank B	15.60 ± 1.01 mm/s^2^
SD. LA Bank B	13.59 ± 0.94 mm/s^2^
LA_0‐40_ Bank B	0.56 ± 0.09
LA_40‐80_ Bank B	0.10 ± 0.04
LA_80‐120_ Bank B	0.04 ± 0.01
LA_120 ‐160_ Bank B	0.08 ± 0.03
LA_160‐200_ Bank B	0.21 ± 0.05
Contrast	27,018.56 ± 3,982.25
Correlation	−0.01 ± 0.02
Energy	6.08 × 10^–5^ ± 2.71 × 10^–5^
Entropy	1.50 ± 0.31
Homogeneity	0.03 ± 0.01

### 2.4 Predictive model generation

#### 2.4.1 Data preparation

The training model dataset included 21 features (16 features from leaf speed and acceleration parameters and 5 features from texture analysis parameters) used as the predictors. The class of GPR was used as the response. The model was separated into two different gamma criteria; 2%/2 mm with a 10% threshold and 3%/2 mm with a 10% threshold. Cross‐validation with five folds was used to protect against the overfitting effect.

The model was trained using two machine learning methods: classification and regression. For the classification model, GPR was classified into the “PASS” and “FAIL” using the institutional warning level. Per the AAPM Task Group 218 recommendation, the tolerance levels of the patient‐specific QA results were set to 95% GPR for the 3%/2 mm criteria.^6^ However, in our clinical experience, most of the patient‐specific VMAT QA in the head and neck seldom exceeded the recommended tolerance level. Therefore, we set the institutional warning level for patient‐specific VMAT QA in the head and neck using average GPR from the previous 657 portal dosimetry measurements, including 214 portal dosimetry measurements from training dataset in our institute. Selecting the average GPR for the institutional warning level forces the system to have high error detection sensitivity. In the training process, this warning level intends to collect the failure population approximately 50%, which can increase the model sensitivity. The institutional warning level was found at 93.70% and 96.53% for the 2%/2 mm and 3%/2 mm criteria, respectively. If the measured GPR exceeds the institutional warning level, the data will be labeled “1” or “PASS.” On the other hand, the measured GPR is lower than the institutional warning level; the data will be labeled “0” or “FAIL.” In training and testing dataset, the abnormal GPR (out‐of‐control) was removed from the dataset using statistical process control^32^ to improve the model accuracy. The lower control limit from I‐chart was used to determine the out‐of‐control GPR, which can be calculated using Equation (3):

(3) $$\text{lower}\;\text{control}\;\mathrm{limit}\;=\;\text{center}\;\text{line}-2.66\cdot \bar{mR},$$

where the center line is averaged GPR, and $\bar{mR}$ is moving range can be calculated using Equation (4):

(4) $$\;\bar{mR}=\;\frac{1}{n-1}\sum_{i\;=\;2}^{n}\left|x_{i}-x_{i-1}\right|,$$

where *n* is the measurement total number, and *x* is individual GPR.

From our QA measurements with portal dosimetry, the lower control limit was calculated, and found at 87.31% and 92.69% for gamma criteria 2%/2 mm, and 3%/2 mm, respectively.

Five out‐of‐control plans were removed from the training and testing dataset with GPR less than the lower control limit for both gamma criteria. The population of the pass and fail the institutional warning level in the training dataset can be summarized as follows: for the gamma criteria of 2%/2 mm with a 10% threshold, the pass and fail the institutional warning level was 49.53% (106 arcs) and 50.47% (108 arcs), respectively; for the gamma criteria of 3%/2 mm with a 10% threshold, the pass and fail the institutional warning level was 57% (122 arcs) and 43% (92 arcs), respectively. The average GPR for the training dataset was 96.53% ± 1.90% for gamma criteria of 3%/2 mm, and 93.50%±2.49% for gamma criteria of 2%/2 mm.

#### 2.4.2 The ensemble of trees model

Our study proposes using only feature extracted from MLC effect and texture analysis for training the predictive model; the ensemble of the trees model in classification and regression methods was selected to train the model because they showed good performance results for the less feature used. The AdaBoost (adaptive boosting) was used for the classification method and fine‐tune with the following hyperparameters; learning rate = 0.1, the number of learners = 500. For the regression method, the bagged ensemble of regression trees was used and fine‐tune with the following hyperparameters; learning rate = 0.1, the number of learners = 500, minimum leaf size = 8.

### 2.5 Model accuracy

In this study, model accuracy was investigated using the testing dataset. The population of the pass and fail the institutional warning level in the testing dataset can summarize as follows: for the gamma criteria of 2%/2 mm with a 10% threshold, the pass and fail the institutional warning level were 68.52% (37 arcs) and 31.48% (17 arcs), respectively; for the gamma criteria of 3%/2 mm with a 10% threshold, the pass and fail the institutional warning level was 59.26% (32 arcs) and 40.74% (22 arcs), respectively. The average GPR for testing dataset was 96.53%±1.30% for gamma criteria of 3%/2 mm, and 93.76%±3.12% for gamma criteria of 2%/2 mm.

The model accuracy was investigated in terms of sensitivity and specificity. The sensitivity and specificity scores were calculated as shown in Equations (5) and (6), respectively. The agreement of failing the institutional warning level between the prediction and measurement was identified as a true positive (TP), while the disagreement was identified as a false positive (FP). The agreement of pass the institutional warning level between the prediction and measurement were identified as true negative (TN), while disagreement were identified as a false negative (FN).

(5) $$\mathrm{Sensitivity}\;\left(\%\right)\;=\;\frac{\mathrm{TP}}{\left(\mathrm{TP}+\mathrm{FN}\right)}\times 100\%,$$

(6) $$\mathrm{Specitivity}\;\left(\%\right)\;=\;\frac{\mathrm{TN}}{\left(\mathrm{TN}+\mathrm{FP}\right)}\times 100\%.$$

The sensitivity represents the probability of the model to detect a failure warning level. Alternatively, the specificity represents the probability of the model to detect a pass warning level. The sensitivity and specificity were also determined for the regression model; however, the model results were predicted as the GPR value. Hence, GPR was classified as a pass or fail the institutional warning level before calculation sensitivity and specificity using the institutional warning level as explained in the predictive model generation session. In addition, the accuracy of the GPR prediction in the regression model was determined using two metrices; the difference between prediction and measurement QA results and root mean square error (RMSE).

## 3 Results

The feature importance was also determined as shown in Table 4. For the AdaBoost classification model, the five most important features at gamma criteria 3%/2 mm were energy, LA_160‐200_ Bank A, LS_12‐16_ Bank B, Mean LS Bank A, and Mean LA Bank B; the five most important features at gamma criteria 2%/2 mm were LS_0‐4_ Bank B, LA_0‐40_ Bank A, entropy, LS_12‐16_ Bank B, and homogeneity.

**TABLE 4:** Ranking of five relative feature importance for different models

	AdaBoost classification		Bagged regression trees	
Five feature rank	Gamma3%/2 mm	Gamma2%/2 mm	Gamma3%/2 mm	Gamma2%/2 mm
1	Energy	LS_0‐4_ Bank B	LA_160‐200_ Bank A	LS_0‐4_ Bank B
2	LA_160‐200_ Bank A	LA_0‐40_ Bank A	SD. LA Bank B	Mean LA Bank A
3	LS_12‐16_ Bank B	Entropy	LS_4‐8_ Bank B	LA_0‐40_ Bank A
4	Mean LS Bank A	LS_12‐16_ Bank B	Mean LS Bank B	Entropy
5	Mean LA Bank B	Homogeneity	LA_160‐200_ Bank B	LA_160‐200_ Bank B

For the bagged regression tress model, the five most important features at gamma criteria 3%/2 mm were LA_160‐200_ Bank A, SD. LA Bank B, LS_4‐8_ Bank B, Mean LS Bank B, and LA_160‐200_ Bank B; the five most important features at gamma criteria 2%/2 mm were LS_0‐4_ Bank B, Mean LA Bank A, LA_0‐40_ Bank A, entropy, and LA_160‐200_ Bank B.

### 3.1 Model accuracy

**FIGURE 2:**
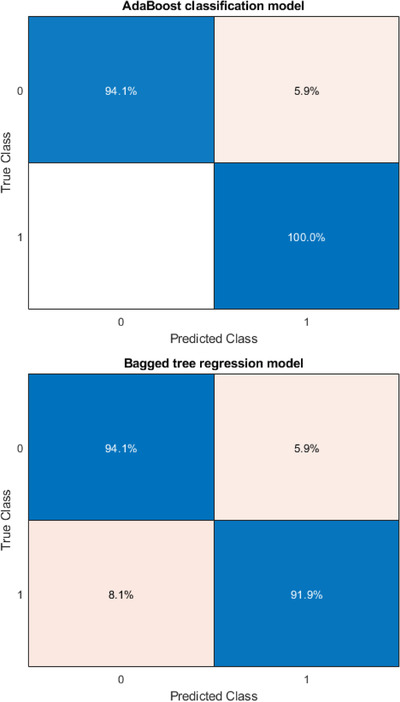
Confusion matrix of AdaBoost classification and bagged tree regression model for gamma criteria of 2%/2 mm

**FIGURE 3:**
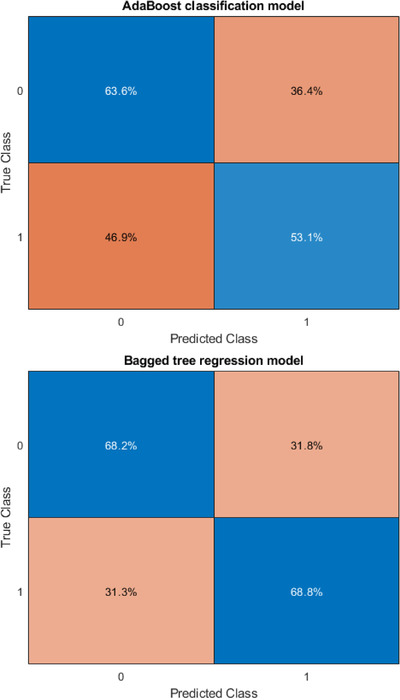
Confusion matrix of AdaBoost classification and bagged tree regression model for gamma criteria of 3%/2 mm

Table 5 summarizes the results of the sensitivity and specificity of the testing dataset for the classification and regression models with different gamma criteria (2%/2 mm with a 10% threshold and 3%/2 mm with a 10% threshold). For gamma criteria of 2%/2 mm with a 10% threshold, the sensitivity of 94.12% was observed in both of bagged regression trees and AdaBoost classification model, while the highest specificity of 100% was observed with the AdaBoost classification model. For gamma criteria of 3%/2 mm with a 10% threshold, the highest sensitivity of 68.18% was observed with the bagged regression trees model, while the highest specificity of 68.75% was observed with the bagged regression trees model. Figures 2 and 3 show the confusion matrix in a different model for gamma criteria of 2%/2 mm and gamma criteria of 3%/2 mm, respectively.

**TABLE 5:** Summary of the sensitivity and specificity in the testing dataset for classification and regression models with two gamma criteria (2**%**/2 mm with a 10**%** threshold, and 3**%**/2 mm with a 10**%** threshold)

Model	AdaBoost classification		Bagged regression trees	
gamma criteria	Sensitivity	Specificity	Sensitivity	Specificity
	(TP/FAIL number)	(TN/PASS number)	(TP/FAIL number)	(TN/PASS number)
2%/2 mm with a 10% threshold	94.12% (16/17)	100% (37/37)	94.12% (16/17)	91.89% (34/37)
3%/2 mm with a 10% threshold	63.63% (14/22)	53.13% (17/32)	68.18% (15/22)	68.75% (22/32)

**FIGURE 4:**
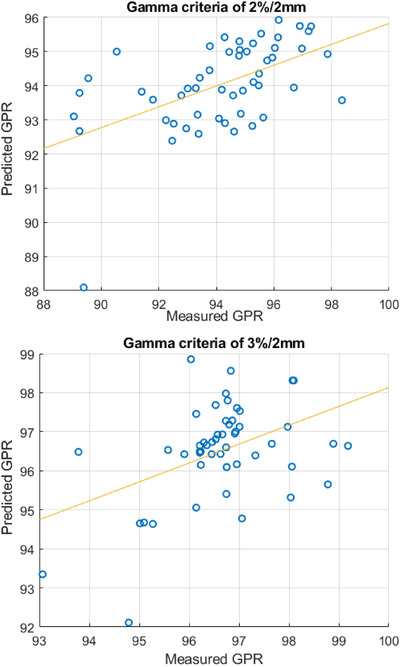
Comparison between measured and predicted GPR in gamma criteria of 2%/2 mm and gamma criteria of 3%/2 mm

In addition, the accuracy of the bagged regression trees model was evaluated using the difference between predicted and measured GPR. For gamma criteria of 2%/2 mm, 85% of prediction is within ± 3% differences, and the RMSE was 2.44. For gamma criteria of 3%/2 mm, 98% of prediction is within ± 3% differences, and the RMSE was 1.22. Figure 4 shows the relationship between measured and predicted GPR for gamma criteria of 2%/2 mm and gamma criteria of 3%/2 mm.

## 4 DISCUSSION

Most of the feature top‐five ranking for both models and both gamma criteria were found at leaf speed and acceleration parameters. Only three features extracted from texture analysis (energy, entropy, and inhomogeneity) were top‐five ranking, implying that the feature extracted from leaf parameters was more important than the feature extracted from texture analysis.

For the tighter gamma criteria of 2%/2 mm, a similar feature top‐five ranking was observed in the different models; the first ranking was found the same feature at LS_0‐4_ Bank B, while the feature of LA_0‐40_ Bank A, entropy, LS_12‐16_ Bank B were found at both models. The agreement between the AdaBoost classification and the bagged regression trees model at the first ranking could imply that the LS_0‐4_ Bank B was the strong correlation between this feature and GPR results. The feature importance results can demonstrate that feature extracted from the MLC effect, and texture analysis was enough to predict the QA results.

Note that the accuracy of the predictive model in our study was improved by implementing the statistical process control^32^ in the pre‐training process. In the pre‐training process, the abnormal (out‐of‐control) GPR values were removed from the training and testing dataset using a lower control limit as calculated from the statistical process control method.

The intention of this system is not to replace the standard patient‐specific QA, but rather to evaluate robustness in order to minimize the likelihood of re‐optimization at a later phase of the treatment planning and preparation process. Patient‐specific QA measurement should still be performed to confirm accuracy and deliverability of the final optimized plan.

For the classification model, the setting of pass and fail tolerance levels influence the model accuracy as described by Valdes et al.^19^ and Tomori et al.^33^ A large number of fail tolerance levels (TP) can increase the model accuracy to predict the fail tolerance level. Because of the small data size in our study, the universal tolerance level recommended by the AAPM Task Group No. 218^14^ cannot be used to classify the pass and fail tolerance level. Therefore, our study used the institution warning level to improve the model accuracy that can collect more population in failing the institutional warning level. Our study was compared with Jiaqi et al.^16^ that had a large population. They observed the highest sensitivity using the random forest (RF) classification model with 66.67% sensitivity (4/6) by setting the action limit to 90% GPR for the gamma criteria of 3%/2 mm, and 100% sensitivity (5/5) by setting the action limit to 80% GPR for the gamma criteria of 2%/2 mm. The highest specificity was observed in the Poisson Lasso (PS) regression model with 100% specificity (42/42) for the gamma criteria of 3%/2 mm, and 43/43 for the gamma criteria of 2%/2 mm. Unlike in our study, the highest sensitivity of 68.18% (15/22) was observed in the bagged regression trees model by setting warning level at 96.53% GPR for the gamma criteria of 3%/2 mm, and sensitivity of 94.12% (16/17) was observed in both bagged trees regression and AdaBoost classification model by setting warning level at 93.7% GPR for the gamma criteria of 2%/2 mm. The highest specificity of 68.75% (22/32) was observed in the bagged regression trees model for the gamma criteria of 3%/2 mm, and specificity of 100% (37/37) was observed in the AdaBoost classification model for the gamma criteria of 2%/2 mm. This difference could be contributed by various factors such as the size of the pass and fail the institutional warning level population in the training and testing dataset, the difference in models used for training, and GPR settings for classification.

Kruse et al.^34^ indicated that the sensitivity to detect error can be further explored using tighter criteria. Similar to this study, the gamma criteria of 2%/2 mm has greater sensitivity than the gamma criteria of 3%/2 mm as demonstrated by the 94.12% and 68.18% sensitivity, respectively, in the classification model, and 94.12% and 63.63% in the regression model.

The advantage of the predictive model in patient‐specific QA is it aids medical physicists to evaluate the risk of plan fails QA. If there is a risk, the medical physicist can re‐optimize the plan by changing a plan parameter such as the MLC speed and acceleration, and variance of intensity fluence (contrast, correlation, energy, entropy, and homogeneity in texture analysis). Additionally, the predictive model can reduce the iterative process, that is, re‐measurement if a failed QA result, which can decrease the change in patient scheduling delay as reported by Abolaban et al.^15^ Moreover, the predictive model can be implemented in the online‐adaptive radiation therapy workflow because the patient‐specific QA process cannot be performed before delivering the beam to the patient due to the time limitations. In online adaptive radiation, only independent dose calculations can be performed before treatment. Other solutions, such as the transit dose measurement^35^, ^36^ and log‐file analysis,^37^ can be used to monitor the dose delivery during treatment. There is no additional process to predict the risk of failed QA results for online adaptive radiation therapy; therefore, the predictive model can solve this problem.

As Vial et al.^38^ reported, disagreement between the EPID measurement and PDIP can be caused by various variables such as differences between the profile correction at the EPID calibration and PDIP, and if the change in the beam spectrum from MLC attenuation does not consider the PDIP. Therefore, this error may affect the accuracy of the model. Vlades et al.^17^ also validated a machine learning approach for predicting GPR using different QA devices, particularly diode‐array detectors and portal dosimetry, and determined that the accuracy of the prediction model at diode‐array detectors was greater than the accuracy at the portal dosimetry (3% compared with 3.5% accuracy) because the portal dosimetry had large disagreements in the low‐dose regions. The EPID measurement in this study was performed using an integrated mode with rotated gantry during measurement to consider the MLC error from the sagging effect, and the IsoCal systems on the Varian TrueBeam was used to correct imager sag during gantry rotation as previously described by Gao et al.^39^

## 5 CONCLUSIONS

The feasibility of the developed model to predict patient‐specific QA of head and neck VMAT plans was demonstrated based on the MLC effect and texture analysis using a machine learning approach. The promising result was found at tighter gamma criteria 2%/2 mm with 94.12% sensitivity (both bagged regression trees and AdaBoost classification model) and 100% specificity (AdaBoost classification model). This tool would reduce the number of re‐QA measurements or re‐plan during the patient‐specific QA process. Future studies will include an implementation method to control beam complexity scores in the optimization and the dose calculation process to reduce the risk of failed QA results.

## CONFLICT OF INTEREST

The authors declare that there is no conflict of interest that could be perceived as prejudicing the impartiality of the research reported.

## AUTHOR CONTRIBUTIONS

All listed authors contributed substantially to the study design, execution, and manuscript drafting and review. All authors approved the final version of the manuscript.

## Data Availability

The data that support the findings of this study are available from the corresponding author upon reasonable requests.
